# Lectures on New Remedies and Their Therapeutical Applications

**Published:** 1863-07

**Authors:** Samuel R. Percy

**Affiliations:** Professor of Materia Medica and Therapeutics


### LECTURES ON NEW REMEDIES AND THEIR THERAPEUTICAL APPLICATIONS.

DELIVERED AT THE
NEW YORK MEDICAL COLLEGE AND CHARITY HOSPITAL.
By SAMUEL R. PERCY, M.D. Professor of Materia Medica & Therapeutics.

### ON THE USE OF VERATRUM VIRIDE AS A MEANS OF ARRIVING AT A CORRECT DIAGNOSIS IN DISEASES OF THE HEART AND LUNGS.

Reprinted from the "American Medical Times."

Gentlemen:—I purpose to-day to relate to you the symptoms I found present in a case of disease of the heart. I will then give you the treatment I adopted for their amelioration, and, as far as time will permit, explain to you the action of the remedy used, and how, by this action, a clear and correct diagnosis could be determined, when, previous to this administration of the remedy, it was almost impossible to arrive at an accurate diagnosis.

James Cunningham, æt. 40, a day-laborer. About four years ago he had acute rheumatism: since then he has complained of impeded and difficult respiration, accompanied with more or less palpitation. These symptoms have increased in severity, until at present he is hardly able to move. The first inspection of this man's face gives one a thrill of pain, for intense suffering is so plainly imprinted upon it: the eyes have a wild and anxious look, the mouth is partly opened, the nostrils are dilated; and these and other marked alterations from the aspect of the features while at rest, are all produced by one necessity, that of better respiration. In a word, we have dyspnœa. If we stop to count the breathing, we find he has from forty-seven to fifty respirations in a minute, and we see that this dyspnœa differs greatly in character from the dyspnœa of asthma or pneumonia; it is rather of a gasping, strangling character. The throat and chest are bare, the arms are rested and poised so as to give the muscles of the chest every opportunity to perform their functions. As you watch him, you see that he makes no effort at motion, or rather that he tries to avoid making the slightest effort, for fear it will increase his dyspnœa; that he even avoids speaking, and looks to others to answer questions for him. Sick as this man looks and feels, he is not in bed, but is seated in a large arm-chair, with his feet upon a pillow, and we learn, upon inquiry, that he has not lain down, or hardly been out of that chair for a week, and that during that time he has scarcely slept for a minute; that although intensely sleepy, the minute his eyes close in sleep he awakes with a sudden start, and a gasp as if suffocation were imminent. As we sit quietly watching him for a few minutes, we see a drowsiness gradually creeping over him, we see his eyelids close, and for a moment or two we can fancy his breathing easier, and he looks as though he might sleep, but in an instant he starts and gasps for breath, and again that look of the horror of suffocation overspreads his face.

We see, then, that in addition to dyspnœa, or difficulty of breathing, he has *orthopnœa*, an inability to assume a recumbent posture during sleep without producing a struggle for breath.

Let us now attend to the state of the pulse. The moment the finger is applied to his radial artery, we find the pulse is a most peculiar one. Instead of the steady beat we find in health, we here have what is usually called a jerking or leaping pulse. It feels as though the impetus given had not been completed, and as two or three fingers are spread over any of the larger arteries, there is a serpentine, wriggling sensation conveyed to them, and this sensation, which may be felt, may be plainly seen, if any of the arteries, either large or small, be closely watched, and it will then be noticed that the arteries have assumed a very tortuous appearance. The bowels are costive, the urine is secreted in small quantities, and it is of a dark red color, containing large quantities of purpurate of ammonia. There is a dry, teasing, irritative cough. The feet and legs are much swollen, and we learn that the swelling has much increased within the last few days, and that it has progressed upwards. What do all these symptoms tell us? To a junior student they explain little of the cause of the disease, but to one of experience every symptom is full of information! The peculiar, serpentine, wriggling pulse, that I have described, is always indicative of one peculiar disease of the heart, and wherever you find this pulse you may safely pronounce that there is regurgitation,—aortic regurgitation. How shall we prove this to be the fact in this individual case? You will say, that ascultation and percussion will plainly settle this point! As with difficulty we get the man into such a position as to listen to the heart, we are struck with the tumultuous amalgamation of sounds and murmurs, and with the closest intensity and nicest perception we are utterly unable to state positively what we do hear. With a rapid respiration of fifty in the minute, and a pulsation too fast to count, how is it possible to arrive at anything like a correct diagnosis? We plainly hear an unnatural murmur, but it is utterly impossible to define its character, or tell with which sound of the heart it occurs.

It is, precisely, in this state of disease that the medicine that I have mentioned, veratrum viride, is of such inestimable value to us, not only in ameliorating the symptoms, but in enabling us to arrive at a correct diagnosis. As this man is in a critical condition, and as the medicine we propose giving him is a powerful sedative, it will be necessary to give it with caution, and watch the state of the pulse from hour to hour. I will commence with a dose of three minims of the concentrated tincture, the formula for which I will give you hereafter. Upon returning in an hour, although the pulse cannot be counted, it is evidently more regular than before, the respirations are now forty-two in the minute, and the patient thinks he feels a little easier. We now give him two minims every hour, for three successive hours, when we see him again. The pulse can now be counted, 132 beats in the minute, but it requires great attention, or you easily loss the count; the respiration is certainly much easier, and is thirty-seven in the minute. The man says, he is already easier than he has been for a week, but he dares not trust himself to sleep for fear of the orthopnœal paroxysm. Leaving him now in the care of an intelligent friend, we shall not see him again till morning. As we see him at 10 A.M., eighteen hours since the administration of the first dose of veratrum viride, we find a very marked change. We learn that he took two minims every hour until midnight; he then felt a little nausea; and he has taken two minims every two hours since midnight. The pulse has gradually decreased in frequency, being now about ninety in the minute, and the respirations thirty-one. Since midnight he has slept at intervals of fifteen to twenty minutes at a time, and wakes up with a struggle. He has also stood on his feet several times to have his cushion shaken up; the urine has been passed in much larger quantity. I now direct that four minims of the tincture, combined with one-eighth of a grain of sulphate of morphia, be given him at 10, 11, and 12 o'clock, and I will see him again before 1 o'clock. You will find, as you use veratrum viride more frequently, that you will occasionally need to give it in full doses without inducing nausea, and, with this object in view, you will combine it with morphia. The morphia again has another adjuvant action: it lessens the number of respirations, when given in combination with veratrum, more readily than either remedy will do alone. And now, at 1 o'clock, in what state do we find our patient? He still sits erect in the chair, but his head has fallen back, and he is sound asleep; the respirations are only twenty-two in the minute, and the irregular pulse, taking an average of three minutes, beats only fifty-five in the minute. Let us now gently awaken him, and examine the state of his heart and lungs by auscultation. We now find a very marked difference from the tumultuous sounds heard at our last examination, for as then all was indistinct and confused, now every sound and murmur can be distinctly appreciated with the greatest ease. There is no difficulty for the youngest student to now readily study and comprehend every normal and abnormal sound. We find, upon percussion, that there is marked hypertrophy of the heart, and that this hypertrophy is general, and has caused a downward subsidance of the organ, and, as is common with dyspnœa from other causes, there is a descent and flattening of the diaphragm. We find, upon further examination, that the severe dyspnœa has caused lung inflation or distention, and that the border of the lung overlaps the heart, which is another cause of downward subsidence. We now plainly hear a distinct murmur and regurgitation following the incomplete closure of the aortic valves, and as the movements of the heart, and the respirations also, are now slow, this imperfect closure of the valves, with the sudden and jerky flow of blood into a partially collapsed aorta, and subsequent regurgitation of blood into the ventricle, with the damming back of the whole current of the circulation, are plainly audible. A murmur is distinctly heard in the carotids, and the serpentine, wriggling movement, of which I have before spoken, can be most easily seen and felt in the arteries that approach the surface. I must not be too minute in my description of pathological conditions, but confine myself to my proper sphere,—the action of medicines. But I must give a brief description of the result of the treatment in this case. You will remember that, previous to the administration of the veratrum, we could hear but little by listening to the lungs. Now, we plainly distinguish that the dyspnœa which exists is not dependent upon want of air supplied to the lungs, but on want of proper circulation within the pulmonary vessels. We spoke of an irritating dry cough. The cough still continues, but not so incessantly as when we first noticed it; and it is not now dry, but there is quite free expectoration of viscid mucus; there is no pus with it, and the complete absence of pus alone is a strong symptom to assure us that no inflammatory action of the lungs was the cause of the dyspnœa, but mere passive congestion, caused by sluggishness of the circulating fluid. The kidneys now have secreted very large quantities of fluid, and it is of a brighter yellow color. This is not because we have given diuretics, but owing to the relief we have given to the circulation, for previously the slow and imperfect passage of the blood through the kidneys prevented the draining off of the proper quantity of water, partly, because of the non-renewal of fresh blood to the kidneys in sufficient quantity to part with its water, and partly because the heart and lungs had not sufficiently metamorphosed or vitalized the blood to present it to the kidneys in quality to be eliminated.

We find our patient much relieved in all his symptoms. The dyspnœa is much relieved; the orthopnœa, for the present, has left him; the cough is less frequent, and, if you watch him, you see that it now scarcely troubles him if he does cough; the urine is free in quantity; he can move, and complains of feeling hungry; and all this relief has been brought about in twenty-one hours by the administration of thirty-seven minims of my concentrated tincture of veratrum viride! Now, how has this small quantity of medicine produced this amelioration? We have frequently before explained to you that veratrum viride is the best arterial sedative that we possess; that when judiciously administered, it regulates the action of the heart, and brings it to its normal standard. It lessens the irritability of the whole vascular system, and causes the blood to flow more readily and quietly. It does this not only by its action on the heart, but, as we have demonstrated by its action on the bloodvessels and upon the blood itself. Its sedative action upon the bloodvessels I have demonstrated in many instances, and I have witnessed a marked change in the character of the blood during the action of this remedy. These peculiar changes in the action of the heart and bloodvessels, and the alteration in the character of the blood, by veratrum viride, I must leave till another lecture. The action we notice in the case I have related is, a gradual subsidence in the rapidity of the circulation, and, consequently, a great relief from the oppressive dyspnœa. As the circulation becomes more quiet, we plainly notice a more thorough contraction of the aortic valves, and, although we have not in any way cured the organic lesion, we have, to a very marked extent, relieved the functional disturbance. And not only have we relieved our patient from intense suffering, but (whereas, when we first saw him it was utterly impossible, by ascultation or percussion, to form any diagnosis as to the extent or character of his disease,) we can now, while he is under the influence of our remedy, form a clear, accurate, and correct diagnosis, without difficulty and without danger.

I have, since 1856, been in the habit of preparing every patient, whose heart and lungs I have wished to examine, with small and proper doses of veratrum viride, and by this means I have been enabled to arrive at a clear and certain diagnosis of cases of incipient phthisis, pleuritis, pneumonia, diseases of the heart, etc., that I could not clearly diagnose without the previous preparation of the patient with this remedy, owing to functional disturbances or other exciting causes. There are many persons who are examined for these diseases, where it is almost impossible to arrive at any correct diagnosis in the early stages of disease, at which time *only* treatment can be expected to be of much avail, owing to even slight functional disturbances, which completely mask or render obscure the signs that without the disturbing causes would be readily recognized. Now, veratrum viride quiets these functional disturbances, lessens the rapidity of the circulation, tranquillizes the respiration, and thus so moderates these functions that the mind can readilly define and arrange the sounds that are communicated to the ear. I give you this new means of diagnosis as the result of my own investigations. I am not aware that it has ever been practiced, except by those to whom I have communicated it. I need not impress upon you its vast importance, for, by means of this practice, you may always know what you are treating, and you will find that that is no slight gain in your ability to inform your patient of what he may expect from your treatment. This new means of diagnosis will be of inestimable value to the Life Insurance Companies in all cases of doubtful diseases of the chest.

But let me, in a few words, finish what I have to say on the treatment of the patient before us, and I must leave further discussion of the interesting subject matter before us to another lecture.

As soon as our patient had entirely overcome all feelings of nausea, half a grain of elaterium was administered to him. It produced a large watery evacuation, and greatly relieved the œdematous condition of the legs. By small doses of veratrum viride, cautiously administered whenever dyspnœa became troublesome, by the admistration of half a grain of elaterium every third day, and by the use of the vegetable tonics, and a nutritious, but carefully watched diet, our patient is out and about his ordinary occupation, but he has to be very careful, or the orthopnœal struggles prevent him from sleeping at night. He will, probably, die suddenly. I have merely related this case as a means of interesting you in the new method of diagnosis I have proposed to you. It was more easy for me to bring it before you in this way.

Of the concentrated tincture of which I have spoken, I have found that which is usually sold in the drug stores under the name of Norwood's Tincture, of very uncertain strength, scarcely ever being alike in two different stores; and I think a great deal of the want of uniformity complained of with this remedy, is owing to the imperfect manner in which the tincture I have spoken of is made. From the many experiments I have performed, I have found that the medicinal principle of the root is contained in the resin. To obviate all difficulties of the uncertainty of strength, I have prepared the tincture I have been in the habit of using, after the following formula, and have always found it uniform in strength:—

CONCENTRATED TINCTURE OF VERATRUM VIRIDE.

Any quantity of well-selected root is coarsely powdered, and treated with alcohol 86°, by percolation, the alcohol is distilled off, and the residuum evaporated to an extract over a water-bath until it is nearly dry, or until it ceases to become lighter upon being weighed at intervals of an hour or two. To make the tincture, one part of this extract is dissolved in ten parts of alcohol at 86°, and filtered.

Any good pharmaceutist can prepare this tincture, but if any of you wish to use it immediately, either the tincture or the extract can be obtained from Mr. Faber, Sixth Avenue, corner of Thirty-eighth Street.

This tincture is nearly double the strength of that called Norwood's, and the medium dose is about two minims. I also use the pure resinoid, and a tincture prepared from it, of which I will speak at another time.

